# Evaluation of Rice Traits Containing H9N2 Subtype Avian Influenza HA Protein Before Commercialization

**DOI:** 10.3390/cimb47120986

**Published:** 2025-11-26

**Authors:** Hongyan Chu, Zhen Hao, Lei Zhang, Yanyue Lou, Yupeng Hua, Wenming Gao, Fei Li, Lichuang Han, Shuangli Bian, Wenbo Cheng, Jiangnan Zhang, Yi Zhu, Shiyuan Pan, Erqin Zhang, Xuannian Wang, Gaiping Zhang

**Affiliations:** 1 International Joint Research Center of National Animal Immunology, College of Veterinary Medicine, Henan Agriculture University, Zhengzhou 450046, China; chy1356184758@163.com (H.C.);; 2 Longhu Laboratory of Advanced Immunology, Zhengzhou 450046, China; 3 School of Advanced Agricultural Sciences, Peking University, Beijing 100871, China; 4 Institute for Animal Health, Henan Academy of Agricultural Sciences, Zhengzhou 450002, China

**Keywords:** rice endosperm expression, genetic stability, agronomic trait, biosafety, plant-derived vaccine

## Abstract

The H9N2 avian influenza virus (AIV) is difficult to prevent and control because of its low pathogenicity and frequent mutation. In a previous study, the HA (hemagglutinin) protein of H9N2 was expressed in a rice endosperm reactor and prepared into a subunit vaccine to immunize chickens and mice, both of which exhibited a good immunity effect. The results of the intermediate tests of the transgenic strains (AIV-1 and AIV-3) showed that the HA gene can be stably expressed. Agronomic traits, such as plant height and number of grains, were significantly optimized in the transgenic strains. Moreover, no exogenous HA genes were found in the leaves of the weeds, and it was initially determined that there was no risk of gene drift. This study provides key technical support for the commercialization of plant subunit vaccines for avian influenza viruses.

## 1. Introduction

H9N2 is a low-pathogenicity avian influenza virus that has seriously endangered the global poultry industry. From 1994 to the present, it has been mutating as a zoonotic disease in China, seriously endangering the poultry industry and human lives and health [1]. In particular, genetic rearrangement or recombination events between H9N2 viruses and other influenza viruses have made it possible for avian influenza viruses to cross the species barrier to infect humans and other mammals, posing a new threat to global public health [2,3,4,5]. Current vaccine technology is facing multiple challenges. For example, the inactivated whole-virus vaccine is the most widely used H9N2 vaccine, which has high safety and low production cost and the vaccine strains that are already on the market in China are all isolated viruses in culture [6,7]; however, facing the ever-mutating influenza viruses, the inactivated vaccine has a long research and development cycle, and is not able to provide multidimensional immunoprotection [8]. The cost of production of subunit vaccines based on baculovirus expression systems is relatively high [9]. Therefore, the urgency of developing a new generation of vaccine technology has been highlighted.

A landmark achievement of modern medicine, vaccines have always been a central pillar of the disease prevention and control system. However, due to many factors, such as high cost, cold chain transportation, and insufficient popularity of traditional vaccines in the production process, plant vaccines, an innovative breakthrough in the field of biotechnology, are gradually becoming an important trend in global public health reform with their unique advantages [10,11,12,13,14,15,16,17,18,19,20,21,22,23,24]. Exogenous proteins synthesized in plants do not contain human and animal pathogens, have a relatively high yield, can be stored in seeds, and have low production costs. Among plant expression systems, rice endosperm has been proved to be an ideal carrier for the production and storage of high-value active substances [16,20,21,23]. It has been successfully used to produce recombinant proteins such as HSA, lactoferrin, and bioactive substances like carotenoids, etc. Previously, we used a rice endosperm reactor to express the E2 protein of classical swine fever [25] and HN protein of Newcastle disease [26], and both of them showed good immunity effects after immunization. On the basis of this, we also expressed the HA antigen of avian influenza virus, H9N2 subtype, and carried out a single immunization on SPF chickens. The results showed that the effective antibody level could be maintained for more than 105 days (unpublished data). Based on this, rice endosperm has the ability of eukaryotic post-translational modification, which can protect the natural structure of antigens and ensure the correct expression of antigenic epitopes.

This study was dedicated to breaking through the technical bottleneck of traditional vaccine production and innovatively developed plant-derived vaccine technology based on a rice endosperm expression system. This technical solution has a triple-innovation value: firstly, it establishes a new environmentally friendly system for avian influenza prevention and control, with a lower production cost than traditional methods and without the need for cold-chain transport; secondly, it creates a technical paradigm for the application of plant bioreactors in the field of livestock and poultry vaccines; and more importantly, it builds a cross-innovation platform between botany and veterinary medicine and provides a replicable technological pathway for the development of livestock and poultry vaccines. The commercial application of plant vaccines must be based on rigorous scientific and systematic evaluation. The actual value of transgenic crops mainly depends on the three core dimensions of genetic stability, agronomic traits, and field biosafety. This breakthrough not only solves the “bottleneck” problem of vaccine production, but also creates a new paradigm for the industrialization of agricultural biotechnology and provides an “approach” for the innovation of the global vaccine production system.

## 2. Materials and Methods

### 2.1. Plant Materials and Measuring Tools

In this study, the HA protein of H9N2 was successfully constructed and expressed using a rice endosperm reactor. After a long period of screening, two transgenic rice strains, AIV-1 and AIV-3, were obtained. AIV-1 and AIV-3 belong to independent transgenic rice strains [27,28,29]. (data on the construction of rice-derived HA and related immunological evaluations have not been published, but for the immunological evaluation of chickens, refer to the PhD thesis of Yanan Wang from Jilin University, and for the evaluation of its immunity in mice, refer to the MSc by Xiangxiang Niu from Henan Agricultural University) All the experimental data were generated based on AIV-1 and AIV-3. Taipei 309 (TP309) was used as a control variety. EACKER straightedge (product 713319 3m) and electronic vernier calipers (model DL91150) were used as the measuring tools.

### 2.2. DNA Extraction from Rice Leaves and Spikes

Panicle tissues were randomly collected from transgenic rice strains (generations T1–T3) and TP309 rice plants at the anthesis stage. The collected panicle tissues and weeds samples were snap-frozen in liquid nitrogen, transported to the laboratory on dry ice, and stored at −80 °C for DNA extraction. Weeds around the experimental fields were collected and preserved using the same method. For each generation, 6 samples were collected for panicles of different transgenic rice varieties and for weeds from different areas around the corresponding paddy fields. DNA samples were extracted from flowering rice spike tissues of generation T1–T3 transgenic rice strains and TP309 plants, which were stored at −80 °C. DNA extraction was performed using a FastPure^®^ plant DNA isolation mini kit (DC104-01, Vazyme, Nanjing, China) following the manufacturer’s instructions. The extracted DNA was stored at −20 °C. DNA from surrounding weeds was extracted using the same procedure. PCR analysis was used to detect the inheritance of the target gene. Primer sequences specific to the HA gene are listed in Table S1, and the PCR system is shown in Table S2. The optimal reaction conditions for PCR were as follows: (1) pre-denaturation at 95 °C for 5 min; (2) 35 cycles of amplification at 90 °C for 30 s, 60 °C for 20 s, and 72 °C for 30 s; (3) final extension at 72 °C for 5 min; and (4) storage at 16 °C. PCR products were analyzed by agarose gel electrophoresis.

### 2.3. Acquisition and qPCR Detection of cDNA in Rice Leaves

Rice leaves of generation T1–T3 plants of AIV-1 and AIV-3 transgenic strains and rice leaves of the TP309 strain were randomly selected at the rice poplar flowering stage, with at least three strains in each group. After the rice leaves were removed, they were immediately snap-frozen in liquid nitrogen, transported from Xinjiang to the laboratory by dry ice transport, and stored at −80 °C for subsequent experiments. Total RNA was extracted using a FastPure^®^ universal plant total RNA isolation kit (RC411-01, Vazyme). cDNA was synthesized via reverse transcription using HiScript^®^ II Q RT SuperMix for qPCR (+gDNA wiper) (R223-01, Vazyme) following the manufacturer’s instructions. The cDNA extracted from leaves of generation T1–T3 transgenic rice and TP309 was used as the template and was amplified by qPCR using primer pairs for the target genes. The primers used are listed in Table S1, and the qPCR system is shown in Table S3. The relative expression levels of the exogenous gene in leaves were determined using the 2^−ΔΔCt^ method using gene eIF4A as an internal reference. At least three replicate experiments were performed for each sample.

### 2.4. Western Blot

Seeds (generation T1–T3 transgenic seeds and TP309 seeds, which were harvested simultaneously and stored under identical conditions were used) from different strains were ground into powder, then mixed with extraction buffer (50 mM Tris with 10 mM NaCl, 1 mM EDTA pH 9.0) at a ratio of 1:5 (*w*/*v*, g/mL). The mixture was stirred for 1.5 h, followed by centrifugation at 9000× *g* for 30 min at 4 °C. The resulting supernatant was collected. Then, 30 μL of the supernatant was placed into 7.5 μL of 5× loading buffer in a 1.5 mLEP tube, boiled in a metal bath at 100 °C for 10 min, and finally centrifuged at 12,000× *g* for 2 min. Pre-prepared 10% protein gel was placed into an electrophoresis tank and 1× SDS electrophoresis buffer solution added, which covered the sample loading hole. The prepared sample was added to each well. An Ar 10–180 kDa prestained protein marker, purchased from Henan Xianyan Biotech Co., Ltd (Zhengzhou, China, ArP01201), and a 180 kDa prestained protein marker, purchased from Vazyme (MP102-01), were used as indicator markers. At 80 V for 30 min, The sample migrates through the concentration gel until it reaches the separation gel. Then, the voltage was adjusted to 120 V for 75 min until the bromophenol blue indicator ran out of the bottom of the gel. Next, transfer printing was carried out to prepare the sandwich. In the order of filter paper–protein gel–nitrocellulose (NC) membrane-filter paper, air bubbles were expelled during the layer–layer accumulation process to ensure that the components were fully bonded. After adding the transfer buffer, transfer was carried out for 95 min under a maximum voltage of 300 V, 250 mA, and constant current conditions. After the transfer was completed, the instrument was removed and the protein membrane placed in an incubation box containing 5% skimmed milk and blocked at room temperature for 1 h. After the blocking had completed, the NC membrane was washed once with TBST and incubated with the H9N2 AIV-positive chicken serum (gifted by Henan Qixiang Biological Technology Co., Ltd (Zhengzhou, China), stored in the laboratory), 500-fold diluted, and used as primary antibody incubate at room temperature for 1 h and washed with TBST three times, each time for 5 min. Subsequently, the NC membranes were incubated with the secondary antibody diluted with 5% skimmed milk (the secondary antibody was HRP, goat anti-chicken IgG purchased from Abbkine (A21080, Wuhan, China), 5000-fold diluted, used as secondary antibody, and incubated at room temperature for 1 h. After being washed with TBST three times, the chromogenic solution (ECL chemiluminescence kit, NCM Biotech, P10100, Newport, RI, USA) was added evenly onto the membrane. Photos were taken and the images saved.

### 2.5. Evaluation of Germination and Seedling Emergence Rate

The materials used for evaluating germination and seedling emergence were generation T1–T3 transgenic strains and TP309 seeds harvested simultaneously and stored under identical conditions. In sum, 100 seeds were randomly taken from each strain, placed in a 37 °C thermostat for 48 h, subsequently transferred to 50 mL conical flasks, and soaked in water at 25 °C in the dark for 36 h. The water was replaced every 12 h to maintain cleanliness. After soaking, the seeds were transferred to 9 cm glass petri dishes lined with moistened sterile filter paper and incubated at 37 °C until radicle emergence. Once more than 80% of the seeds showed radicle protrusion through the seed coat, they were transferred to a 25 °C incubator and moistened regularly to promote germination. Germination was assessed every 12 h and recorded when radicles and shoots were visible. When a shoot reached half the grain length, the seeds were transplanted into moist nutrient soil in the culture room at 28 °C during the day and 25 °C at night, with a 14 h light/10 h dark photoperiod in 65% RH (relative humidity). Seedling emergence data were collected and recorded. Throughout the experiment, the seeds were kept consistently moist. Germination and seedling emergence rates were calculated as percentages. Germination data and seedling emergence data were both recorded consecutively three times, and the experimental results were expressed as percentages.

### 2.6. Developmental Cycle Analysis

The developmental cycle analysis was performed using generation T1–T3 transgenic rice strains and TP309 control seeds harvested simultaneously and stored under uniform conditions. Seeds were sterilized and germinated in medium. Post-germination, seedlings were transferred to a culture room and then potted in soil. After 20 days, the seedlings were transplanted uniformly to the outdoor field in two strains, AIV-1 and AIV-3 (total land length 30 m, width 20 m), with a spacing of 50 cm between the different generations and cultivated under adequate irrigation. The developmental cycle was monitored, tracking both vegetative and reproductive growth stages, with the spikelet stage marking the transition between these phases.

### 2.7. Evaluation of Comprehensive Agronomic Traits

A comprehensive evaluation of agronomic traits related to growth patterns was conducted on transgenic rice strains of the T1–T3 generations at the full maturity stage—which is defined as the period when over 90% of the glumes turn yellow and the basal seeds harden and become resistant to breakage. At this stage, rice spikes from each strain were collected into the corresponding numbered seed bags. After sun-drying for 3 days under outdoor conditions, agronomic traits related to spikes and grains were measured.

The recorded agronomic traits related to growth pattern and their specific definitions were as follows: plant height, defined as the distance from the base of the plant to the tip of the second tallest leaf; effective tillers, referring to the number of tillers bearing spikes with more than five mature seeds, counted from the base upward; flag leaf length, measured from the base to the tip of the flag leaf; flag leaf width, indicating the maximum width of the flag leaf; and single-plant mass, which refers to the mass of the entire plant (with roots) after cleaning and blotting dry with a paper towel to remove surface moisture.

The relevant agronomic traits recorded for rice spikes and their specific definitions were as follows: effective spike number, defined as the number of spikes with more than five mature grains per plant; spike length, the distance from the neck node to the tip of an effective spike; effective spike mass, the mass of the effective spike on a single plant; grain number per spike, the total number of grains in an effective spike; fruiting rate, the percentage of filled grains in an effective spike; grain density, the number of grains per centimeter of spike length; and thousand-grain mass, the mass of 1000 filled grains.

The recorded seed quality traits and their specific definitions were as follows: brown rice percentage, the ratio of brown rice mass (after hull removal) to the total grain mass (before hull removal); grain length, the average length of 10 grains; grain width, the average width of 10 grains; grain thickness, the average thickness of the rice grains; and chalkiness, the proportion of the white, opaque portion in the rice grain, calculated based on the chalkiness rate under fluorescent light and the average area of the chalky portion.

### 2.8. Scanning Electron Microscope Observation of Rice Seeds

Three seeds were randomly selected from each of the different transgenic rice varieties across three generations. Intact grains were gently fractured using tweezers to expose a flat cross section as uniformly as possible. The cross sections were mounted on sample stubs with the exposed surface facing upward and then coated with platinum using an ion sputter coater (Cressington 108 Auto, Cressington Scientific Instruments Ltd., 34 Chalk Hill, Watford WD19 4BX, UK). The morphology and particle size of starch granules were examined using an environmental scanning electron microscope (Model Q45, FEI Company, Hillsboro, OR, USA). Multiple observation areas were randomly selected and imaged.

### 2.9. Identification of Pollen Viability by Iodine–Potassium Iodide Staining Method

At the early flowering stage of transgenic rice strains from the T1–T3 generations, mature anthers were collected from the upper, middle, and lower parts of panicles. Three samples were randomly collected from each variety of transgenic rice strain. These anthers were left at room temperature for 0 h, 3 h, and 6 h, respectively, then transferred onto microscope slides. Each anther was gently crushed with forceps, and 1–2 drops of 1% I–KI solution were added using a Barton’s dropper to fully release the pollen grains. A coverslip was placed over the sample and gently pressed with forceps, followed by 2–3 min staining. A 10× microscope objective was used to observe randomly selected fields to examine anther morphology and record the proportion of mature anthers. The results were used to assess differences in pollen viability between transgenic strains and the recipient variety.

### 2.10. Field Biodiversity Evaluation

Field insect diversity survey: When the T3 generation rice cultivated outdoors reached the milky stage, three consecutive rain-free days were selected for sampling. Sticky traps were placed around each rice strain plot, with three random sampling points per strain and two sticky traps at each point. After three days, the sticky traps were collected and the trapped insects identified and counted to provide a preliminary assessment of pest presence during the reproductive growth stage. Field plant diversity survey: For the rice cultivated outdoors, plant diversity was surveyed at the tillering stage (immediately after transplanting) and at maturity. At each stage, 3–5 plots per strain were randomly selected. All plants within a 0.25 m^2^ area (50 cm × 50 cm) surrounding each plot were collected, and their species diversity and biomass were recorded to preliminarily assess changes in plant diversity before and after planting of the transgenic materials.

### 2.11. Statistical Analysis

Data are presented as means ± standard error of the mean (SEM). All experiments were conducted under a single-variable design, and *p* values were calculated using ordinary one-way analysis of variance (ANOVA) with α set to 0.05. All experiments were set up with at least three biological replicates. All data were verified using GraphPad Prism version 8.0 and met the normality of the data and equal variances tested. All graphs were generated using GraphPad Prism version 8.0.

## 3. Results

### 3.1. Stable Expression of Ha Antigen in Different Generations of Rice

To test the stability of the H9N2 rice-derived HA protein in transgenic rice strains AIV-1 and AIV-3, we planted and followed up their T1–T3 generations to monitor their genetic stability.

**Figure 1:**
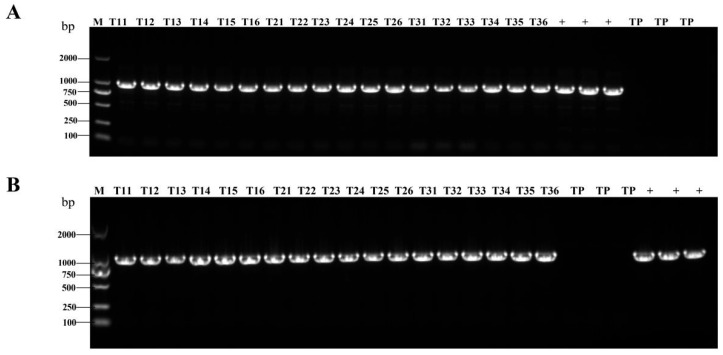

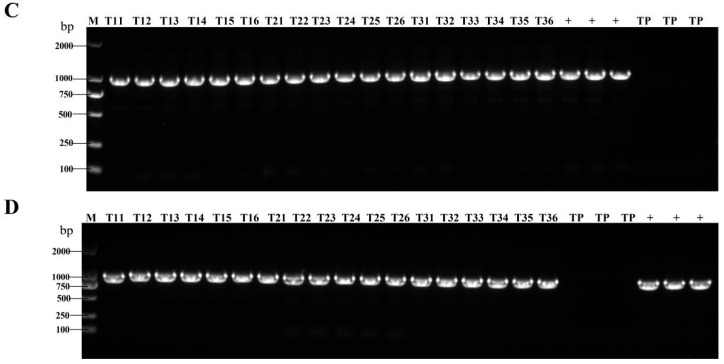
Stable presence of HA gene in transgenic rice detected by PCR across generations. (**A**,**B**) PCR (amplicon size: 945 bp) was used to detect HA genes in rice leaves at the flowering stage and in rice spikes in different generations from AIV-1. M is marker size, which refers to the size of the DNA fragments; T11–T16 denote six randomly selected rice strains from generation T1, T21–T26 denote six randomly selected rice strains from generation T2, T31–T36 denote six randomly selected rice strains from generation T3, TP indicates TP309, + indicates positive plasmid. (**C**,**D**) PCR (amplicon size: 945 bp) was used to detect HA genes in rice leaves at the flowering stage and in rice spikes in different generations from AIV-3.

**Figure 2:**
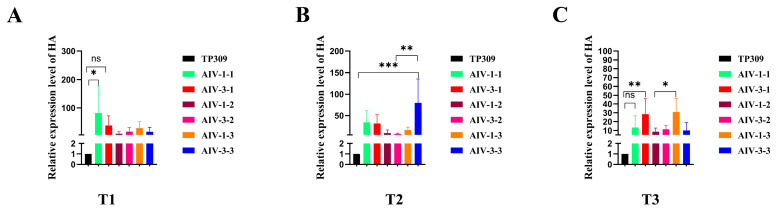
HA gene in the leaves of transgenic rice at the flowering stage by q-PCR. (**A**–**C**) T1, T2, and T3 generations, respectively. AIV-1 and AIV-3 are screened rice plants that were both successfully transfected with the exogenous HA gene and expressed it successfully; AIV-1-1, AIV-1-2, and AIV-1-3 refer to three biological replicates randomly selected by AIV-1; AIV-3-1, AIV-3-2 and AIV-3-3 refer to three randomly selected biological replicates of AIV-3 (three randomly selected biological replicates for each generation). These data were statistically significantly different from those above on one-way ANOVA: * *p* < 0.05, ** *p* < 0.01, and *** *p* < 0.001.

**Figure 3:**
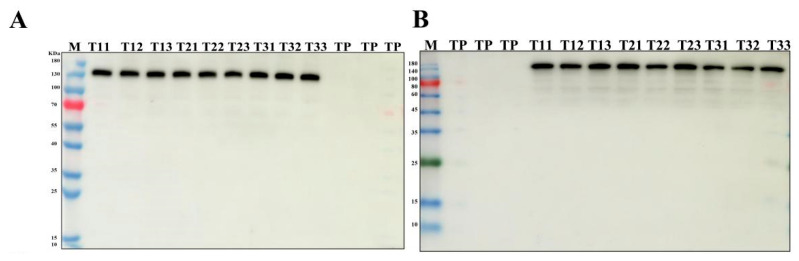
Protein-level detection of HA protein expression in rice seeds of different generations. (**A**,**B**) Expression of HA gene in rice seeds of different generations of AIV-1 and AIV-3 by Western blot. T11–T13 denote three randomly selected rice strains from generation T1, T21–T23 denote three randomly selected rice strains from generation T2, and T31–T33 denote three randomly selected rice strains from generation T3.

AIV-1 and AIV-3 grown in the field were selected and their rice leaves and spikes were randomly collected during the flowering stage of rice. PCR detected the presence of the HA genes in both leaves (Figure 1A,C) and spikes (Figure 1B,D). qPCR detected the transcript level of HA in the leaves, which was low, but present (Figure 2A–C). Western blot detection of protein levels in rice of different generations showed that HA protein was stably expressed in rice seeds of different generations (Figure 3A,B). The results indicated that HA protein was stably expressed in different generations of rice at the DNA level, the mRNA level, and the protein level, and HA protein was stably present in different generations of rice.

### 3.2. Combined Agronomic Traits of Generation T1–T3 Transformant Strains and TP309

**Figure 4:**
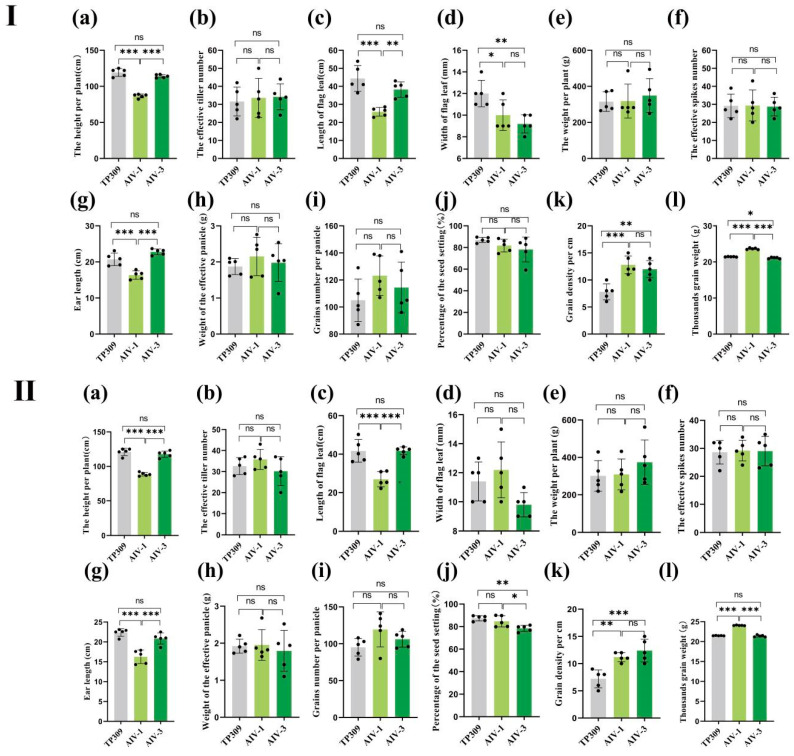

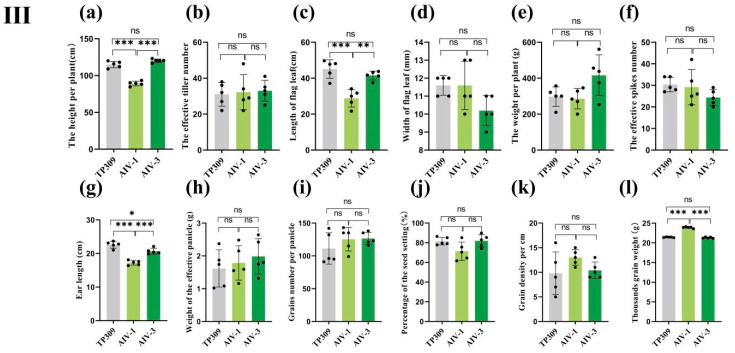
Combined agronomic traits of generation T1–T3 (AIV-1 and AIV-3 of generations T1–T3 correspond to (**I**–**III**)) transformant strains and low gluten. On 12 comprehensive agronomic traits recorded in generations T1–T3—(**a**) height per plant, (**b**) effective tiller number, (**c**) length of flag leaf, (**d**) width of flag leaf, (**e**) mass per plant, (**f**) effective spike number, (**g**) ear length, (**h**) mass of effective panicle, (**i**) grain number per panicle, (**j**) percentage of seed setting, (**k**) grain density per cm, and (**l**) thousand-grain mass—the data were statistically significantly different from the above data on one-way ANOVA: * *p* < 0.05, ** *p* < 0.01, and *** *p* < 0.001. Both AIV-1 and AIV-3 were assessed with five randomly selected strains.

During rice development, yield-related factors can be categorized into growth morphology, panicle status, and grain quality. We observed agronomic traits related to these three factors in rice generations T1–T3. Furthermore, in the T1, T2, and T3 generations, the plant height, length of flag leaf, and ear length of the AIV-1 strain were lower than those of TP309, while in the T1 and T2 generations, the grain density and thousand-grain mass of the AIV-1 strain were higher than TP309, and in the T3 generation of AIV-1, only the thousand-grain mass of the AIV-1 strain was higher than TP309. Furthermore, the AIV-3 strain exhibited reduced flag leaf width and thousand-grain mass compared to TP309, but higher grain density in generation T1 (Figure 4I). In the T2 generation, the AIV-3 strain showed slight reductions in percentage of seed setting (Figure 4II). In the T3 generation, the ear length of AIV-3 was slightly lower than that of TP309 (Figure 4III). The plant height, flag leaf length, and ear length of AIV-1 were lower than AIV-3, while the thousand-grain mass of AIV-1 was significantly higher than that of AIV-3. The plant height, flag leaf length, and ear length of AIV-1 were lower than those of AIV-3, while the thousand-grain mass of AIV-1 was higher than that of AIV-3 in the T1,T2, and T3 generations (Figure 4III), In the T2 generation, the percentage of seed setting of AIV-1 was higher than that of AIV-3.

### 3.3. Grain Phenotypes of Generation T1–T3 Transformant Strains and Tp309

**Figure 5:**
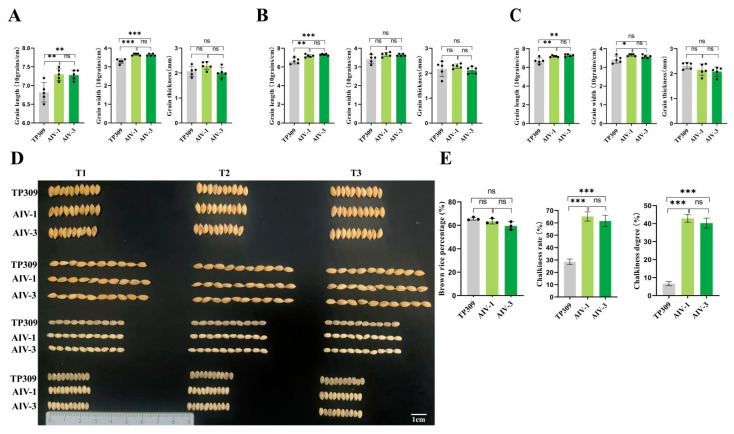
Grain phenotypes of generation T1–T3 transformant strains and low gluten. (**A**–**C**) Comparison of rice grain morphology data for each strain in the T1, T2, and T3 generations. From left to right: grain length, grain width, and grain thickness (both AIV-1 and AIV-3 were assessed with five randomly selected strains). (**D**) Rice grain phenotypes (brown rice on top, fine rice on bottom, *n* = 10). (**E**) Comparison of rice grain quality among the strains. From left to right, in order of brown rice percentage, chalkiness and chalkiness (*n* = 3). The data were statistically significantly different from the above data on one-way ANOVA. ns, no significant difference; * *p* < 0.05; ** *p* < 0.01; *** *p* < 0.001.

In the T1 generation (Figure 5A), the grain length and width of transgenic rice strains AIV-1 and AIV-3 were significantly increased compared to TP309. The grain thickness showed no significant change compared to TP309. In the T2 generation (Figure 5B), the grain length of AIV-1 and AIV-3 was significantly increased compared to TP309, with no significant difference between the two strains. The grain width and thickness showed no significant changes compared to TP309. In the T3 generation, the grain length of AIV-1 and AIV-3 was significantly increased compared to TP309. The grain width of AIV-1 was increased compared to TP309, while that of AIV-3 showed no significant change. The grain thickness of both strains showed no significant change compared to TP309 (Figure 5C). The grain length and width of brown rice (upper) and milled rice (lower) are shown (Figure 5D). Moreover, the Brown rice rate, chalkiness rate, and chalkiness degree are also important characteristics of rice strains (Figure 5E). The brown rice rate showed no significant difference among the three strains, while the chalkiness rate and chalkiness degree of AIV-1 and AIV-3 were significantly increased compared to TP309.

### 3.4. Scanning Electron Microscope Observation of the Internal Structure of Tp309, AIV-1, and AIV-3

**Figure 6:**
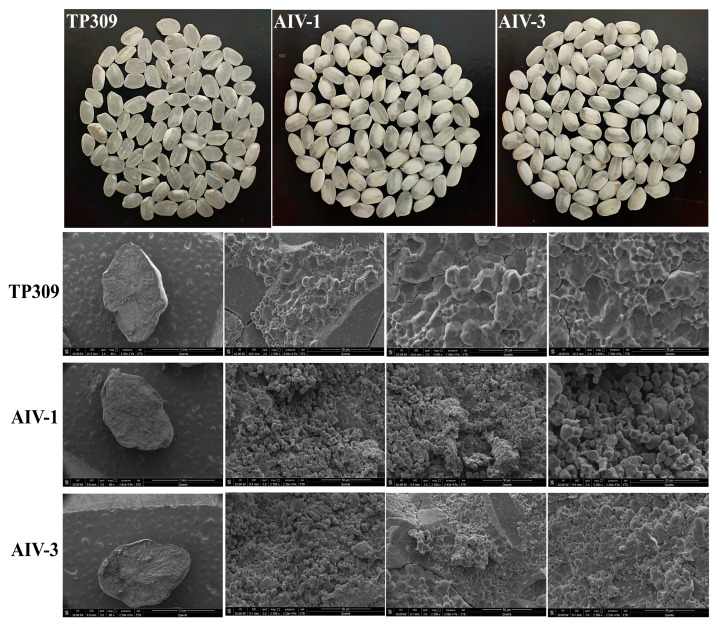
Scanning electron microscope detection of TP309, AIV-1, and AIV-3 rice grain chalkiness (*n* = 3).

To further confirm the chalkiness of the grains, we observed the internal starch arrangement using a scanning electron microscope (Figure 6).

The results showed that the AIV-1 and AIV-3 strains had increased branched-chain starch, decreased straight-chain starch, and a relatively loose starch structure resulting in more brittle grains compared with TP309. These results suggest that the overall agronomic traits remained favorable. Chalkiness and chalkiness were significantly higher in AIV-1 and AIV-3, and the grains were more brittle. This is a key advantage for efficient protein extraction by mechanical milling. In view of these characteristics, AIV is particularly promising for the industrial production of plant-derived vaccines through grain processing.

### 3.5. Germination and Seedling Percentage Assessment of AIV-1 and AIN-3 Transgenic Rice

To determine whether transgenic rice strains AIV-1 and AIV-3 affected seed germination-related traits, seeds harvested were screened and germinated using the filter paper culture method.

**Figure 7:**
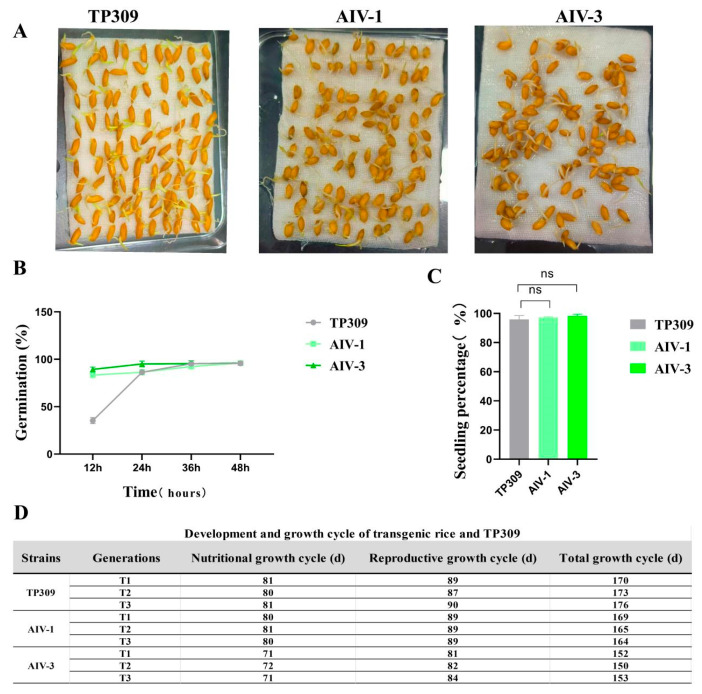
Germination and seedling percentage assessment of AIV-1 and AIV-3 transgenic rice. (**A**) Visualization of germination rates of AIV-1, AIV-3, and TP309. (**B**,**C**) Germination and seedling percentage of transgenic rice (*n* = 100). (**D**) Development and growth cycle of transgenic rice and TP309. The data were statistically significantly different from the above data on one-way ANOVA, where “ns” indicates no significant difference.

The germination data were recorded every 12 h four consecutive times, and the results showed that there was no statistically significant difference between AIV-1 and AIV-3 and TP309 (Figure 7A). At 12 h, 24 h, 36 h and 48 h, the germination rate of TP309 was a little later compared to that of AIV-1 and AIV-3, but the final germination rate was almost the same (Figure 7B). Subsequently, seedlings were grown by the soil pot method and emergence data were recorded. The seedling emergence rate reached 90% (Figure 7C). The two transgenic lines, AIV-1, and AIV-3, showed no significant difference in total growth cycle compared with TP309 plants. To investigate whether there were differences in growth and development between the physiologically improved rice strains AIV-1 and AIV-3 and TP309 plants, the transgenic strains and TP309 were cultivated outdoors for three consecutive generations, and their growth cycles were followed and recorded. The total growth cycle of the TP309 and transgenic strains was relatively stable, fluctuating within the range of 150–170 days (Figure 7D). For the AIV-1 and AIV-3 strains, the asexual growth cycles of the T1, T2, and T3 generations were comparable to those of the wild type and the reproductive growth cycles. These results indicate that under nutrient-sufficient conditions, transgenic AIV-1 and AIV-3 rice had total growth cycles comparable to TP309.

### 3.6. Comparison of Pollen Viability Between Transformant Strains of Generations T1–T3 and Tp309

**Figure 8:**
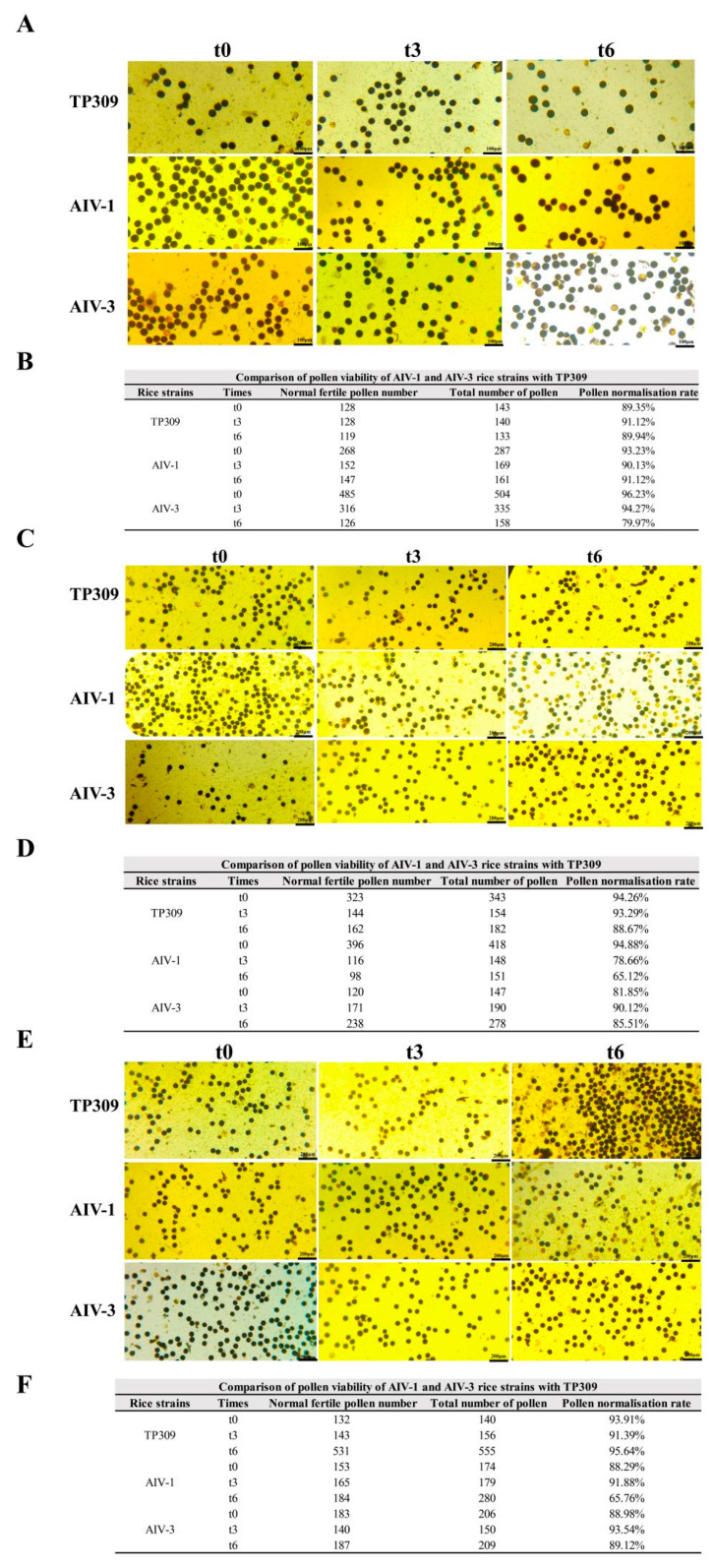
Comparison of pollen viability between transformant strains of generations T1–T3 and TP309. (**A**,**B**) Comparison of pollen viability of generation T1 transformant strain and TP309, respectively. (**C**,**D**) Comparison of pollen viability of generation T2 transformant strain and TP309, respectively. (**E**,**F**) Comparison of pollen viability of generation T3 transformant strain and TP309, respectively.

Rice generally produces seeds through self-pollination, and its rice pollen viability is closely related to its reproductive ability, which directly affects the survival competition between rice plants and other surrounding plants. We assessed the competitiveness of transgenic rice strains for survival under field conditions by staining mature anthers at the primordial stage with potassium iodide. In the T1 generation, the normal pollen rate was 89.35%, 91.12%, and 89.94% for TP309, 93.23%, 90.13% and 91.12% for AIV-1, and 96.23%, 94.27%, and 79.97% for AIV-3 at 0 h, 3 h and 6 h, respectively (Figure 8A,B). In the T2 generation, the normal pollen rate was 94.26%, 93.29%, and 88.67% for TP309 and 94.88%, 78.66%, and 65.12% for AIV-1 at 0 h, 3 h, and 6 h, respectively. In the T2 generation, the normal pollen ratio was 81.85%, 90.12%, and 85.51% for AIV-3 at 0 h, 3 h, and 6 h, respectively (Figure 8C,D). In the T3 generation, TP309 was 93.91%, 91.39%, and 95.64% for T3 at 0 h, 3 h, and 6 h, respectively, 88.29%, 91.88%, and 65.76% for AIV-1, and 88.98%, 93.54%, and 89.12% for AIV-3, respectively (Figure 8E,F). These findings indicate that the pollen longevity and viability of transgenic rice were relatively reduced compared to TP309.

### 3.7. Safety Evaluation of Rice Strains Transgenic for AIV-1 and AIV-3

The environmental safety of genetically modified plants cannot be ignored. Based on this, leaf samples from various weed species were collected from outdoor rice field cultivation plots.

**Figure 9:**
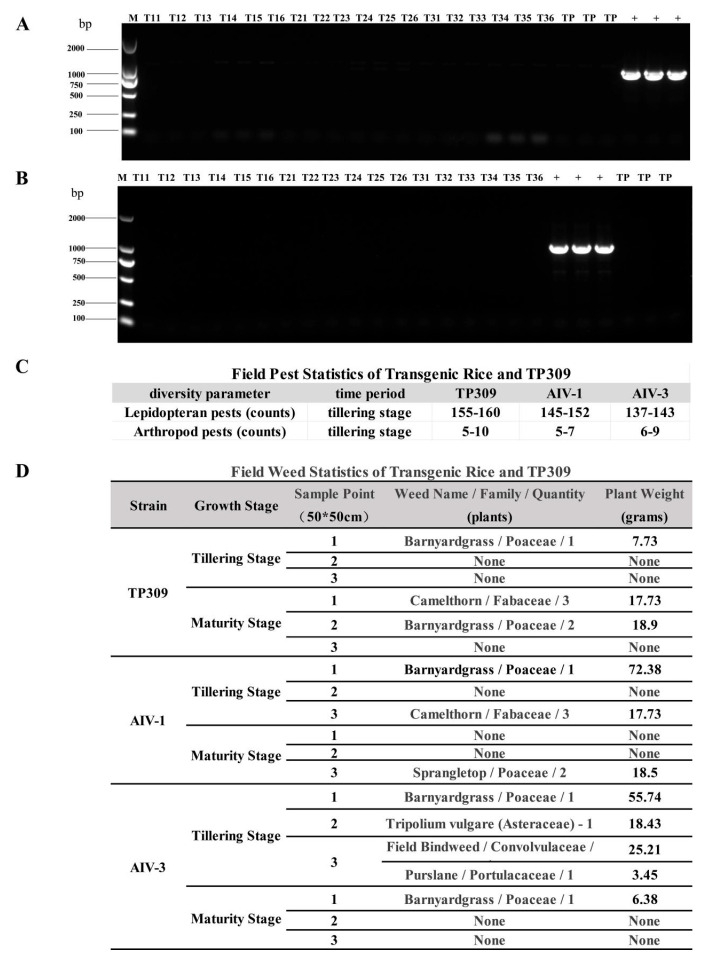
Safety evaluation of rice strains transgenic for AIV-1 and AIV-3. (**A**,**B**) PCR result of the spread of exogenous genes to surrounding weeds from AIV-1 and AIV-3. M is marker size, which refers to the size of the DNA fragments, T11–T16 denote six randomly selected rice strains from generation T1, T21–26 denote six randomly selected rice strains from generation T2, T31–36 denote six randomly selected rice strains from generation T3, TP indicates TP309, and + indicates positive plasmid. (**C**,**D**) Outdoor field biodiversity analysis of transgenic rice and TP309.

The presence of the avian influenza HA gene in non-rice plants was determined via PCR using HA-specific primers (Table S1). We found no evidence of gene transfer in the collected weed samples. Only the positive plasmid showed a band containing the exogenous gene HA, and no gene transfer was found in the other weeds collected (Figure 9A,B). In order to evaluate whether transgenic rice would affect the population and quantity of other surrounding organisms in the field, the experiment on insect diversity selected mildly ripe rice with more serious insect infestation, placed armyworm plates within the range of wild-type and transgenic rice blocks (50 cm × 50 cm), and the quantity of lepidoptera and arthropod insects on the armyworm plates after recovery was observed on the third day. The results showed that the number of lepidoptera insects around the rice planting area was much greater than that of arthropods, but there was no significant difference between the species and number of insects in the TP309 field and the transgenic AIV-1 and AIV-3 field (Figure 9C). In the plant diversity experiment, TP309 and transgenic strains were randomly sampled and the species of all other plants in the block during transplantation and harvesting were recorded. The results showed that compared with the TP309 control, the species of weeds around transgenic strains AIV-1 and AIV-3 had no significant impact (Figure 9D). It can be preliminarily concluded that transgenic rice has little effect on the biodiversity of the surrounding environment. The combined data of reduced pollen viability and absence of transgene spread support the environmental safety of these strains for agricultural use, addressing key regulatory concerns for transgenic crops.

## 4. Discussion

The avian influenza virus H9N2 belongs to the Orthomyxoviridae family of influenza A viruses, whose genome consists of eight segmented single-stranded negative-stranded RNAs that mediate infection of host cells through haemagglutinin (HA) and neuraminidase (NA) [8,9,30].

Although classified as low-pathogenicity avian influenza (LPAI), H9N2 viruses can enhance cross-species transmission through the HA protein 226 site mutation (Leu→Gln), and have developed endemic epidemics in countries such as China and Egypt [31,32,33,34]. Infected birds mainly exhibit respiratory symptoms, decreased egg production, and secondary bacterial infections, resulting in heavy annual losses in the global poultry industry [30]. Existing prevention and control mainly rely on inactivated vaccines, but the traditional chicken embryo culture process carries the risk of antigenic drift [35,36,37,38,39]. In contrast, plant-based bioreactor systems, particularly rice endosperm expression systems, offer a promising alternative due to their low production costs, high biosafety, and breakthroughs in complex cold-chain dependencies and transport requirements.

The results of previous animal experiments showed that the HA protein of the H9N2 avian influenza virus expressed in rice endosperm provided good immunity to both chickens and mice. Animal studies showed that two doses of 0.5 μg and 1 μg of a rice-derived HA vaccine protected mice and chickens, respectively, against lethal attack by the H9N2 virus. After optimizing the immunization regimen, a single dose of 10 μg rice-derived HA vaccine-immunized SPF chickens produced effective antibody levels that lasted for more than 105 days (unpublished data). These results demonstrated that HA proteins produced in rice endosperm have excellent antigenicity and immunogenicity. To further evaluate the feasibility of this vaccine platform, we conducted a comprehensive assessment of genetic stability, agronomic traits, and biosafety of AIV-1 and AIV-3. The results of PCR, qPCR, and Western blot showed that the exogenous HA gene of H9N2 AIV was expressed in different generations of rice. These findings emphasize the genetic stability of the HA protein in the rice endosperm expression system without loss of exogenous genes with planting generations, which is a key prerequisite for its commercial application.

In addition, to assess the effect of transgenes on agronomic traits in rice, we compared the performance of the AIV-1 and AIV-3 transgenic lines with that of the TP309 control. The results showed that the transgenic lines germinated earlier than TP309, but the final germination rate and growth cycle were comparable to TP309. Furthermore, the transgenic lines exhibited comparable or even superior agronomic traits in terms of growth pattern, spike condition, and seed quality. Notably, the AIV transgenic lines not only showed increased grain length and width, but also a significant increase in their powderiness and degree of chalkiness, which was also preliminarily verified by the scanning electron microscopy results for the internal starch percentage. This trait could possibly be explained by our triple-generation sequencing results, which showed that the exogenous gene was inserted into chromosome 1 and chromosome 7 in the genome of TP309 rice. Field trials evaluated the viability and gene transfer potential of transgenic rice strains. Pollen viability analysis showed a slight reduction in pollen longevity and fertility of the transgenic strains compared to TP309. Importantly, PCR analyses of weed samples from paddy fields did not reveal evidence of HA gene transfer, confirming the effective control of the transgene and the environmental safety of the transgenic rice strains. Moreover, insect diversity and plant diversity experiments showed that the transgenic rice had little effect on the biodiversity of the surrounding environment. These findings further support the environmental safety of the rice-derived HA protein production system.

The feasibility of large-scale production of the H9N2 HA vaccine based on the rice endosperm expression system is further highlighted by its excellent performance in genetic stability, agronomic adaptability, and environmental safety. The stable long-term expression of HA protein in transgenic rice seeds (generations T1–T3) combined with the mechanized milling and extraction process can significantly reduce the cost of large-scale production of the vaccine and avoid the risk of antigenic drift in traditional chicken embryo cultures. However, differences in the plant-specific glycosylation pattern from natural viral HA proteins still need to be optimized by targeted modification or adjuvant dosing to enhance neutralizing antibody affinity. In addition, the conclusion of no significant ecosystem impact of transgenic rice in field trials lays a biosafety foundation for subsequent regulatory approval of commercial cultivation.

## 5. Conclusions

This study demonstrated that rice strains (AIV-1 AIV-3) expressing HA proteins of avian influenza viruses maintained good genetic stability through multigenerational field trials. The core agronomic traits of the transgenic rice strains were not significantly different from those of the wild type, but the growth cycle of AIV-3 was shorter than that of AIV-1, and exogenous genes of the transgenic rice strains would not be transferred to the surrounding plants. Overall, this study provides a solid foundation for the commercialization of a rice vaccine against H9N2 subtype HA avian influenza.

## Data Availability

The data from this study are available from the corresponding authors upon request. The restricted availability is due to the authors needing to apply for an environmental release test with these data in order to bring this product to the market and commercialize it.

## Supplementary Materials

The following supporting information can be downloaded at https://www.mdpi.com/article/10.3390/cimb47120986/s1. Table S1: Primers used to amplify HA and eIF4A gene; Table S2: PCR system; Table S3: qPCR system.

## References

[1] Sun Y., Liu J. (2015). H9N2 influenza virus in China: A cause of concern. Protein Cell.

[2] Zhu S., Nie Z., Che Y., Shu J., Wu S., He Y., Wu Y., Qian H. (2024). The Chinese Hamster Ovary Cell-Based H9 HA Subunit Avian Influenza Vaccine Provides Complete Protection against the H9N2 Virus Challenge in Chickens. Viruses.

[3] Zhai K., Dong J., Zeng J., Cheng P., Wu X., Han W., Chen Y., Qiu Z. (2024). Global antigenic landscape and vaccine recommendation strategy for low pathogenic avian influenza A (H9N2) viruses. J. Infect..

[4] Wei Y., Qi L., Gao H., Sun H., Pu J., Sun Y., Liu J. (2016). Generation and protective efficacy of a cold-adapted attenuated avian H9N2 influenza vaccine. Sci. Rep..

[5] Yang W., Dai J., Liu J., Guo M., Liu X., Hu S., Gu M., Hu J. (2022). Intranasal Immunization with a Recombinant Avian Paramyxovirus Serotypes 2 Vector-Based Vaccine Induces Protection against H9N2 Avian Influenza in Chicken. Viruses.

[6] Teng Q., Shen W., Liu Q., Rong G., Chen L., Li X., Chen H., Yang J. (2015). Protective efficacy of an inactivated vaccine against H9N2 avian influenza virus in ducks. Virol. J..

[7] Song C.L., Liao Z.H., Shen Y., Wang H., Lin W.C., Li H., Chen W.G., Xie Q.M. (2020). Assessing the efficacy of a recombinant H9N2 avian influenza virus-inactivated vaccine. Poult. Sci..

[8] Liu S., Zhuang Q., Wang S., Jiang W., Jin J., Peng C., Hou G., Li J. (2020). Control of avian influenza in China: Strategies and lessons. Transbound. Emerg. Dis..

[9] Dong J., Zhou Y., Pu J., Liu L. (2022). Status and Challenges for Vaccination against Avian H9N2 Influenza Virus in China. Life.

[10] Zhu Q., Tan J., Liu Y.G. (2022). Molecular farming using transgenic rice endosperm. Trends Biotechnol..

[11] Zahmanova G., Aljabali A.A.A., Takova K., Minkov G., Tambuwala M.M., Minkov I., Lomonossoff G.P. (2023). Green Biologics: Harnessing the Power of Plants to Produce Pharmaceuticals. Int. J. Mol. Sci..

[12] Yuki Y., Kiyono H. (2009). Mucosal vaccines: Novel advances in technology and delivery. Expert Rev. Vaccines.

[13] Yuki Y., Kiyono H. (2008). [MucoRice: Development of rice-based oral vaccine]. Nihon Rinsho Meneki Gakkai Kaishi.

[14] Wu Z., Zhang Q., Guo Y., Yang H., Yang T. (2022). [Improving the production of plant-based recombinant protein: A review]. Sheng Wu Gong Cheng Xue Bao.

[15] Stander J., Mbewana S., Meyers A.E. (2022). Plant-Derived Human Vaccines: Recent Developments. BioDrugs.

[16] Santoni M., Gecchele E., Zampieri R., Avesani L. (2022). Plant-Based Systems for Vaccine Production. Methods Mol. Biol..

[17] Sang Y., Millwood R.J., Neal Stewart C. (2013). Gene use restriction technologies for transgenic plant bioconfinement. Plant Biotechnol. J..

[18] Nochi T., Takagi H., Yuki Y., Yang L., Masumura T., Mejima M., Nakanishi U., Matsumura A. (2007). Rice-based mucosal vaccine as a global strategy for cold-chain- and needle-free vaccination. Proc. Natl. Acad. Sci. USA.

[19] Lomonossoff G.P., D’Aoust M.A. (2016). Plant-produced biopharmaceuticals: A case of technical developments driving clinical deployment. Science.

[20] Jung J.W., Zahmanova G., Minkov I., Lomonossoff G.P. (2022). Plant-based expression and characterization of SARS-CoV-2 virus-like particles presenting a native spike protein. Plant Biotechnol. J..

[21] Giddings G., Allison G., Brooks D., Carter A. (2000). Transgenic plants as factories for biopharmaceuticals. Nat. Biotechnol..

[22] Fausther-Bovendo H., Kobinger G. (2021). Plant-made vaccines and therapeutics. Science.

[23] Capell T., Twyman R.M., Armario-Najera V., Ma J.K., Schillberg S., Christou P. (2020). Potential Applications of Plant Biotechnology against SARS-CoV-2. Trends Plant Sci..

[24] Azegami T., Itoh H., Kiyono H., Yuki Y. (2015). Novel transgenic rice-based vaccines. Arch. Immunol. Ther. Exp..

[25] Xu Q., Ma F., Yang D., Li Q., Yan L., Ou J., Zhang L., Liu Y. (2023). Rice-produced classical swine fever virus glycoprotein E2 with herringbone-dimer design to enhance immune responses. Plant Biotechnol. J..

[26] Ma F., Xu Q., Wang A., Yang D., Li Q., Guo J., Zhang L., Ou J. (2024). A universal design of restructured dimer antigens: Development of a superior vaccine against the paramyxovirus in transgenic rice. Proc. Natl. Acad. Sci. USA.

[27] Zhao X., Zhang E., Xu Q., Ma F., Wang Y. (2020). Stable and efficient expression and activity identification of avian influenza virus H9N2 subtype HA protein in rice endosperm. Acta Agric. Boreali-Sin..

[28] Niu X. (2021). Preliminary Evaluation of the Immunisation Effect of the HA Protein of Rice-Derived H9N2 Subtype Avian Influenza Virus and Establishment of a Rapid Antibody Detection Method. Master’s Thesis.

[29] Wang Y. (2023). Immunogenicity Study of Haemagglutinin Protein of Avian Influenza Virus Subtype H9N2 Expressed in Rice Endosperm and Identification of B-Cell Epitopes. Ph.D. Thesis.

[30] Peacock T.H.P., James J., Sealy J.E., Iqbal M. (2019). A Global Perspective on H9N2 Avian Influenza Virus. Viruses.

[31] Cao Y., Liu H., Liu D., Liu W., Luo T., Li J. (2022). Hemagglutinin Gene Variation Rate of H9N2 Avian Influenza Virus by Vaccine Intervention in China. Viruses.

[32] Jin H., Wang W., Yang X., Su H., Fan J., Zhu R., Wang S., Shi H. (2018). Evolution of H9N2 avian influenza virus in embryonated chicken eggs with or without homologous vaccine antibodies. BMC Vet. Res..

[33] Chen S., Zhu Y., Yang D., Yang Y., Shi S., Qin T., Peng D., Liu X. (2017). Efficacy of Live-Attenuated H9N2 Influenza Vaccine Candidates Containing NS1 Truncations against H9N2 Avian Influenza Viruses. Front. Microbiol..

[34] Abdi H.M., Mayahi M., Boroomand Z., Shoshtari A. (2021). Avian Influenza-Killed Vaccine on Tissue Distribution and Shedding of Avian Influenza Virus H9N2 in Ducklings. Arch. Razi Inst..

[35] Liu Y., Zhao D., Zhang J., Huang X., Han K., Liu Q., Yang J., Zhang L. (2023). Development of an Inactivated Avian Influenza Virus Vaccine against Circulating H9N2 in Chickens and Ducks. Vaccines.

[36] Lee J., Cho A.Y., Kim D.H., Lee J.B., Park S.Y., Choi I.S., Lee S.W., Song C.S. (2023). Live recombinant Newcastle disease virus vectored vaccine expressing the haemagglutinin of H9N2 avian influenza virus suppresses viral replication in chickens. Avian Pathol..

[37] Ducatez M.F., Becker J., Freudenstein A., Delverdier M., Delpont M., Sutter G., Guérin J.L., Volz A. (2016). Low pathogenic avian influenza (H9N2) in chicken: Evaluation of an ancestral H9-MVA vaccine. Vet. Microbiol..

[38] Cargnin Faccin F., Perez D.R. (2024). Pandemic preparedness through vaccine development for avian influenza viruses. Hum. Vaccines Immunother..

[39] Fellahi S., Nassik S., Maaroufi I., Tligui N.S., Touzani C.D., Rawi T., Delvecchio A., Ducatez M.F. (2021). Pathogenesis of Avian Influenza Virus Subtype H9N2 in Turkeys and Evaluation of Inactivated Vaccine Efficacy. Avian Dis..

