# Supplementary material for: Evaluation of Rice Traits Containing H9N2 Subtype Avian Influenza HA Protein Before Commercialization
Source: Curr Issues Mol Biol. 2025 Nov 26;47(12):986. doi: 10.3390/cimb47120986 (PMC12731790; doi:10.3390/cimb47120986)
Supplement: Supplementary file 1 [file cimb-47-00986-s001.zip › cimb-3964804-supplementary.pdf]

Table S1 Primers used to amplify HA and eIF4A gene

primer	primer sequence（5′-3′）	lengths
HA-F	GAGGGCCTCATCTACGG	945bp
HA-R	GACCTTGCTGGTGATCTTG	
HA-F	GACACCGCCCAGACCAA	229bp
HA-R	TCTCGCCGCTGAGGAT	
eIF4A -F	TTGTGCTGGATGAAGCTGATG	76bp
eIF4A -R	GGAAGGAGCTGGAAGATATCATAGA	

Table S2. PCR reaction system（50μL）

Reaction system	volumes（50μL）
2xRapid Taq master mix (P222-01,Vazyme)	25μL
ddH2O	add to 50μL
HA-F	1μL
HA-R	1μL
cDNA	2μL

Table S3 qPCR reaction system（20μL）

Reaction system	volumes（20μL）
2xChamQ Universal SYBR qPCR Master Mix	10μL
ddH2O	add to 20μL
HA-F	0.4μL
HA-R	0.4μL

cDNA	2μL
